# Development of a Prediction Model to Identify Children at Risk of Future Developmental Delay at Age 4 in a Population-Based Setting

**DOI:** 10.3390/ijerph17228341

**Published:** 2020-11-11

**Authors:** Nienke H. van Dokkum, Sijmen A. Reijneveld, Martijn W. Heymans, Arend F. Bos, Marlou L. A. de Kroon

**Affiliations:** 1 Department of Pediatrics, Division of Neonatology, Beatrix Children’s Hospital, University Medical Center Groningen, University of Groningen, Hanzeplein 1, 9713GZ Groningen, The Netherlands; a.f.bos@umcg.nl; 2 Department of Health Sciences, University Medical Center Groningen, University of Groningen, Hanzeplein 1, 9713GZ Groningen, The Netherlands; s.a.reijneveld@umcg.nl (S.A.R.); m.l.a.de.kroon@umcg.nl (M.L.A.d.K.); 3 Department of Epidemiology and Biostatistics, Amsterdam University Medical Center, location VU, University Medical Center, de Boelelaan 1089a, 1081HV Amsterdam, The Netherlands; mw.heymans@vumc.nl

**Keywords:** prediction model, developmental surveillance, developmental delay

## Abstract

Our aim was to develop a prediction model for infants from the general population, with easily obtainable predictors, that accurately predicts risk of future developmental delay at age 4 and then assess its performance. Longitudinal cohort data were used (*N* = 1983), including full-term and preterm children. Development at age 4 was assessed using the Ages and Stages Questionnaire. Candidate predictors included perinatal and parental factors as well as growth and developmental milestones during the first two years. We applied multiple logistic regression with backwards selection and internal validation, and we assessed calibration and discriminative performance (i.e., area under the curve (AUC)). The model was evaluated in terms of sensitivity and specificity at several cut-off values. The final model included sex, maternal educational level, pre-existing maternal obesity, several milestones (smiling, speaking 2–3 word sentences, standing) and weight for height z score at age 1. The fit was good, and the discriminative performance was high (AUC: 0.837). Sensitivity and specificity were 73% and 80% at a cut-off probability of 10%. Our model is promising for use as a prediction tool in community-based settings. It could aid to identify infants in early life (age 2) with increased risk of future developmental problems at age 4 that may benefit from early interventions.

## 1. Introduction

The World Health Organization estimates that 8% of all children under 5 years have some type of developmental deficit [1]. In the United States, estimates are even higher, with developmental deficits occurring in 15% of children aged 3 to 17 years [2]. In approximately 10% of all children worldwide, developmental deficits occur as early as 4 to 12 months of age [3]. Developmental delay in childhood has been adversely related to academic achievement in adolescence [4] and adulthood [5,6]. Early interventions, i.e., at preschool age, have been shown to improve the functioning of these children [7,8,9], with earlier interventions yielding greater developmental gains [7,8,9]. Several early intervention services have been evaluated in these reviews, mainly preschool programs focusing on language acquisition, motor development, or both [7,8,9]. Effects included enhanced cognitive and motor development in infancy, academic achievement and social competence at school age, as well as a higher socio-economic status (that is higher rates of high school graduation, employment or home ownership) [7,8,9].

The positive effects of early intervention confirm the importance of timely identification of the children concerned. Ideally, such identification should be conducted in a community-based setting, where all eligible children receive follow-up. An example of such an environment is community-based well-child care, such as preventive child healthcare (PCH) offered in the Netherlands. PCH includes repeated consultations, which are offered to all children from birth until adolescence; these consultations are free of charge and attended by over 95% of all Dutch children during the first years of life [10]. Many factors from birth onwards in several domains (i.e., perinatal factors, developmental milestones, or growth) have been associated with developmental delay (i.e., lagging behind in psychomotor, cognitive, or language development from preschool age to adolescence) [4,11,12,13,14,15,16,17,18,19,20,21,22,23]. However, some of these, specifically clinical factors such as results from cranial ultrasound, umbilical cord pH values, or extensive developmental tests, cannot easily be obtained in community-based well-child settings.

A prediction tool could assist clinicians in identifying children at high risk of future developmental delay. However, studies on prediction models for developmental delay in childhood are scarce [24,25,26]. Moreover, most models developed to date require information that is usually not available for all children in community-based settings. To be effective in community-based well-child care, a prediction model and eventually a tool should be based on information commonly recorded or easily obtainable in such settings. Therefore, the aim of our study was to develop a prediction model for infants from the general population, with easily obtainable predictors, that accurately predicts risk of future developmental delay at age 4, and to assess its performance, in order to support well-child care professionals and pediatricians, thereby enhancing possibilities for timely interventions.

## 2. Materials and Methods

### 2.1. Setting and population

For this study, we used data from the Longitudinal Preterm Outcome Project (LOLLIPOP). LOLLIPOP is a community-based cohort consisting of both full-term and preterm (early and moderately-late preterm) children. As preterm children in particular are at greater risk of developmental delay and are well represented in this cohort, the data suited our aim of developing a prediction tool. Thirteen PCH services participated in LOLLIPOP, from which 45,446 children, full-term and preterm (gestational age [GA] < 36 weeks) and comprising almost 25% of all Dutch 4-year-olds, were screened for eligibility. After every second preterm child, the next full-term child from the same birth year was selected as a control. In addition, the cohort was enriched with early preterm children (GA < 32 weeks) from 5 neonatal intensive care units, who were alive upon discharge. Children with major congenital malformations and syndromes were excluded. Eventually, a total inclusion of 2517 children (79%) were achieved in LOLLIPOP. For 1983 children, developmental data within the preset timeframe were available.

### 2.2. Outcome Measure

Development was assessed upon school entry (i.e., at age 4 in the Dutch setting), using the Dutch version of the parent-reported Ages and Stages Questionnaire (ASQ) 48 months’ form. Our research group showed that the Dutch ASQ, although not a diagnostic instrument, is a valid, reliable, cost-effective, fast, and easy way to screen children for developmental delay with a sensitivity to predict special educational needs at age 5 of 89% and corresponding specificity of 80% [26]. The ASQ 48 months’ form contains 30 items in 5 developmental domains: communication, gross motor, fine motor, problem solving, and personal–social functioning [26]. All five domains add up to an ASQ total score, which indicates a child’s general development. A total score below -2 standard deviations (SD) for the reference group of Dutch full-term children was considered to be abnormal and was coded as 1, compared to a normal score, which was coded as 0.

### 2.3. Candidate Predictors

Based on a literature search, we identified predictors associated with developmental delay. To prevent overfitting of the prediction model, we made a selection from these variables, based on their expected predictive strength and feasibility of use in community-based well-child care. We grouped our candidate predictors into four groups: (1) perinatal factors, i.e., GA and birth weight [11,12], sex [13,14,15], being born small-for-gestational-age (SGA) [16], multiple birth [17], and Apgar score [22]; (2) parental factors, i.e., maternal educational level [18], pre-existing maternal obesity [17], and maternal smoking during pregnancy [19] (3) growth, i.e., weight for height z score at 1 and 2 years [20], and (4) developmental milestones, i.e., smiling [21], speaking 2–3 word sentences [5,21], head-lifting [6,21], standing [4,5,21], and walking [5,21].

GA was verified by early ultrasound measures during pregnancy in over 95% of all participants. We extracted information on candidate predictors from a general questionnaire upon inclusion and cross-examined using either PCH or hospital files. We defined pre-existing maternal obesity as a body mass index (BMI) >30kg/m^2^. Maternal educational level was defined as low (i.e., <12 years of formal education) or high (i.e., 12+ years of formal education). Being born SGA was classified as <P10 according to the Dutch Kloosterman curves [27]. Apgar score was assessed 5 min after birth, and a score <7 was classified as abnormal. Growth measurements of height and weight were determined according to protocol at PCH at 1 and 2 years of age. Weight for height z scores were calculated according to the fourth Dutch nationwide growth study [28]. Developmental milestones were assessed in PCH. Smiling is normally achieved at approximately 6 weeks of age, upon which PCH physicians asked parents for the exact age in weeks. The developmental milestones head-lifting (3 months), standing (1 year), walking (18 months), and speaking 2 to 3-word sentences (2 years) were assessed by the PCH physician as attained versus not attained.

### 2.4. Statistical Analyses

Statistical analyses were conducted using R version 3.5.1 (R Foundation for Statistical Computing, Vienna, Austria) and SPSS version 25.0 (IBM Corp., Armonk, NY, USA). All analyses and reporting were conducted according to the Transparent Reporting of a multivariable prediction model for Individual Prognosis Or Diagnosis (TRIPOD) guideline [29]. First, we described data completeness and participant characteristics. Second, we imputed missing values using the Multivariate Imputation by Chained Equations (MICE) method, assuming the data to be missing at random [30], leading to 10 imputed datasets. Then, we used backwards selection to select variables for our prediction model, using a *p*-value of <0.157 (Akaike Information Criterion) [29]. Next, we performed internal validation, using bootstrapping techniques [29], generating 250 bootstrap samples from the original dataset, and applying replacements from the original sample and the prediction model in the bootstrapped samples. Using this procedure, we estimated and corrected for optimism in regression coefficients.

For the prediction model, we used the Hosmer–Lemeshow test to test the agreement between observed and predicted probabilities of developmental delay, interpreting it as a good fit when the *p*-value was above 0.05. We also assessed discriminative performance, both before and after internal validation. Discriminative performance (i.e., the potential of the prediction model to distinguish between children with and without developmental delay) was assessed using the area under the curve (AUC) of the receiver operating characteristic (ROC) curve.

Next, we evaluated our prediction model in terms of sensitivity, specificity, positive predictive value (PPV) and negative predictive value (NPV) at several cut-off values.

Finally, we constructed a tool with the help of Excel, a calculator to automatically determine the probability of an abnormal ASQ score at 4 years of age (available upon request).

### 2.5. Ethical Consideration

Written informed consent was obtained from all parents or guardians of the participants in this study. The study was conducted in accordance with the Declaration of Helsinki, and the protocol was approved by the Ethics Committee of University Medical Center Groningen (METc 2005/130). Clinical Trial registry name and registration number could be accessed on controlled-trials.com, ISRCTN 80622320.

## 3. Results

### 3.1. Population Characteristics

Of all children in our sample, 8.9% had an ASQ total score below −2 SD. Descriptive values of candidate predictors and information regarding missing data are summarized in Table 1. Data completeness for individual variables ranged from 27% to 100%.

### 3.2. Prediction Model

**Figure 1:**
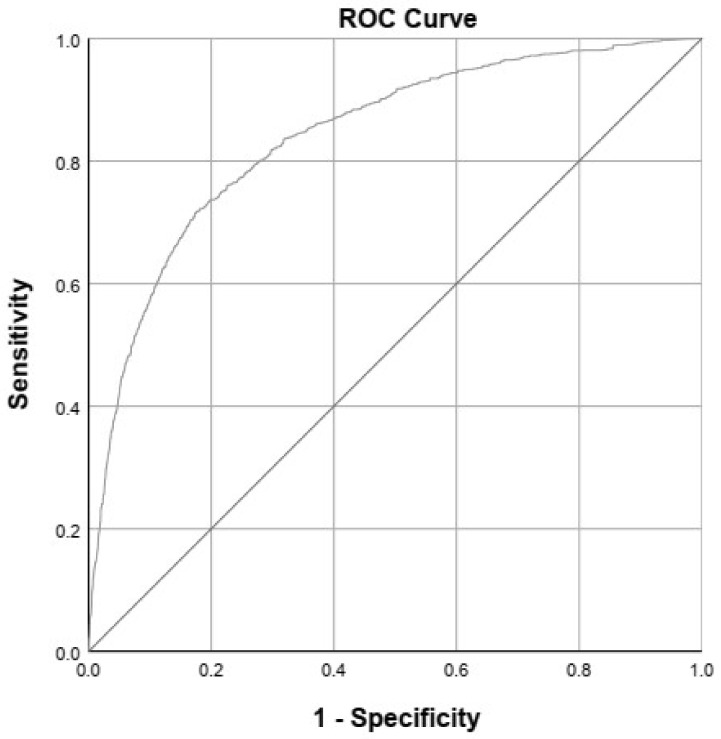
Receiver operating characteristic (ROC) curve after correction for optimism over the 10 imputed datasets (area under the curve = 0.837).

The variables in the final prediction model are presented in Table 2 and consisted of variables from all four categories (i.e., perinatal factors, parental factors, growth and developmental milestones). These variables regard sex, maternal educational level, pre-existing maternal obesity, smiling, speaking 2 to 3-word sentences, standing, and weight for height z score at 1 year. Not attaining speaking 2 to 3-word sentences at the age of 2 years implied a five-fold increase in the likelihood of an abnormal ASQ total score at age 4 (OR 5.45, 95%-CI 3.41–8.69). The model showed good calibration; the fit of the model was good, with a *p* = 0.98 on the Hosmer–Lemeshow test and an explained variance of 31% (Nagelkerke’s R^2^). Our model had a relatively good discriminative performance. Before internal validation, the model showed an AUC of 0.844, and after internal validation (i.e., after correction for optimism in the regression coefficients), the AUC showed only a slight decrease, to 0.837 (Figure 1). In Table 3, we present the evaluation of our prediction model at several probability cut-off values. The sensitivity (73%) and specificity (80%) values were reasonably good when using a cut-off probability of 10% (i.e., a predicted probability of ≥10% is considered “at risk”).

### 3.3. Example

Based on the model, we developed a tool, a calculator in Excel. To illustrate the use of the developed calculator in Excel, we introduced a hypothetical child and assume it to be of male sex, having a mother with an education <12 years and pre-existing obesity, having a BMI z score of −0.09 at 1 year of age, and being behind in development, with a smiling age of 13 weeks calendar age (normally approximately 6 weeks) and non-attainment of standing (normally at approximately 1 year) and speaking (normally at approximately 2 years). According to the calculator, the probability that the child’s ASQ will be abnormal at 4 years of age is 63% (Table 4). If this child were to have had a mother without pre-existing obesity, and if he had attained standing and speaking on time, the predicted probability of an abnormal ASQ score at 4 years of age would already be reduced to 6.8%. The first child would be considered at risk, whereas the second would not.

## 4. Discussion

In this study, we developed a prediction model to predict general developmental delay upon school entry at age 4. With only seven predictors, our prediction model had a good performance. These predictors are easy to obtain in community-based well-child care. The calculator, a tool that was developed on the basis of the underlying model, can support well-child care professionals and pediatricians in identifying children who could benefit from early preventive interventions.

In line with previous studies, we found sex [13,14,15], maternal educational level [17], pre-existing maternal obesity [17], smiling [21], speaking 2 to 3-word sentences [5,6,7,8,9,10,11,12,13,14,15,16,17,18,19,20,21], standing [4,5,21], and BMI z score at 1 year [20] to be predictors. Candidate predictors that did not improve the model were GA, birth weight, being born SGA, being part of a multiple, a low Apgar score, maternal smoking during pregnancy, head-lifting, walking, and BMI z score at 2 years, even though these factors were found to be associated with developmental delay in earlier studies [5,11,12,16,17,19,21,22,31]. Very remarkably, neither GA nor birth weight were included in the final prediction model, although several studies have found prematurity and very low birth weight to be highly predictive of developmental outcomes [11,12,32]. One explanation could be a correlation with included predictors in the final model, although we found no evidence for multi-collinearity. An alternative explanation may be that the developmental milestones are a proxy of a combination of several perinatal variables, such as GA, being born SGA, and perinatal conditions, as the attainment of these milestones is highly related to these variables [33].

We found that our model, based on an inclusion of factors that are easy to obtain in community-based settings, had a good performance, with an AUC of 0.84. We found one comparable prediction model, by Nelson et al., who predicted school readiness on the basis of nine variables, after evaluation of a wide range of predictors obtainable in community-based settings, with an AUC of 0.76 [25]. Importantly, the ASQ and school readiness can be considered as comparable outcome measures, because they both include similar milestones assessed at the same age. However, this study did not include growth and multiple developmental milestones in earlier life as potential predictors. In contrast, two other prediction models that aimed to predict neurodevelopmental outcomes in early infancy included variables that can only be obtained in clinical settings. In the first, Lodha et al. evaluated the Clinical Risk Index for Babies score, including among other factors admission temperature and base-excess determined by blood tests, and they reported good performance, with an AUC of 0.84 [23]. In the second, Yeo et al. constructed a prediction model including among others the use of prenatal steroids, with a much poorer performance, i.e., an AUC of 0.65 [24]. Compared to these clinical prediction models, our prediction model performed relatively well and is of use particularly in community-based settings because of its easily obtainable component variables.

Our prediction model, which has been operationalized in our calculator, showed relatively good sensitivity and specificity at a cut-off predicted probability of 10%. The American Academy of Pediatrics (AAP) has deemed a sensitivity and specificity of around 70% to 80% to be reasonable, even though these values are somewhat lower than generally accepted for medical screening tests, where a specificity of 90% is more common [34]. Routine PCH assessments consisting of the factors that are part of the prediction model we developed are feasible in a population-based setting and only take a little time. With the predictor variables in our model, a risk estimate can be obtained at 2 years of age. Since PCH offers repeated consultations, our model may lead to more intense surveillance in the crucial period between this age and school entry (4 years) in children at increased risk at age 2. Therefore, our model could be used as a tool to aid clinicians in their developmental surveillance, i.e., in making a risk calculation of whether a child is likely to encounter developmental delays later in life.

Our model used the ASQ total score as an outcome measure, which indicates a general developmental delay. Our results might have yielded different predictors regarding the domain scores underlying this total score, i.e., communication, gross motor, fine motor, personal social skills, and problem solving. However, the ASQ total score summarizes development on all these domains and may therefore identify children with delays in any of these domains. An assessment of a high risk of future delay may lead to a more extensive development assessment, e.g., with the Bayley Scales of Infant and Toddler Development, to get a better picture of the development at age 2. Moreover, developmental delay in one domain is often accompanied by a delay in another domain. This focus on general delay aligns with the nature of early intervention services offered to a child, which mostly do not focus on a specific skill (such as motor skills) but rather offer a wide package of support for a child’s development.

### 4.1. Strengths and Limitations

Our study has several strengths. First and foremost, we retrieved this prediction model using a large dataset from a community-based cohort study, which enabled us to consider many candidate predictors and enhanced the applicability of our model in community-based practice. Second, we internally validated our prediction model by using bootstrapping techniques [29]. Therefore, we believe that our correction for optimism is realistic.

We also acknowledge some limitations of our study. First, instead of a neuropsychological test, we used a parent-reported outcome measure, i.e., the ASQ, to determine developmental delay at age 4. However, the ASQ is considered a valid screening tool for the assessment of general developmental delay, with high sensitivity (89%) and acceptable specificity (80%) for the detection of school problems [26]. Second, some of our variables had a relatively high percentage of missing values. This possibly means that relatively little attention was given to these variables during the consultations, although these data are easy to obtain. We were able to limit the influence of these missing values by using multiple imputation techniques. Third, the predictive values that we calculated (i.e., the NPV and PPV) are population specific. The prevalence of developmental delay was relatively high in our population because it included an oversampling of preterm children. Therefore, for a general population, for which this prediction model was developed, the positive predictive value would probably be lower and the negative predictive value would probably be higher. Finally, we did not have information on early intervention services used by the participating children, which may have decreased the predictive power of our model somewhat.

### 4.2. Implications

Our results regarding the performance of our prediction model are promising. However, our model should first be externally validated in another cohort to assess whether adaptations to specific populations are needed. Future studies in this field should examine the possibility of developing a dynamic prediction model [35] that can be applied within PCH, enabling a reassessment of the risk after incorporating new information into the model, which may further improve developmental surveillance. Future studies should also focus on implementing the calculator in practice, e.g., by including the calculator into the digitized (preventive) well-child care files, in several settings, and evaluate the effects of combining the calculator with targeted interventions.

## 5. Conclusions

In conclusion, we developed a prediction model based on only seven easily obtainable variables to predict general developmental delay at age 4 in the general population, with good performance properties. This model is promising for use as a tool in community-based settings, aiding PCH professionals and pediatricians in identifying children at increased risk of developmental delay who may benefit from early preventive interventions.

## Figures and Tables

**Table 1:** Description of candidate predictors for the LOLLIPOP population for analysis (*N* = 1983).

Candidate Predictors	Descriptive Value	Data Available for *n* (%)
*Perinatal Factors*		
Gestational age at birth in weeks, mean (SD)	34.3 (4.05)	1983 (100)
Gestational age 24–32 weeks, *n* (%)	513 (26)	
Gestational age 32–36 weeks, *n* (%)	627 (32)	
Gestational age 37–42 weeks, *n* (%)	543 (27)	
Male sex, *n* (%)	1065 (54)	1982 (99.9)
SGA, *n* (%)	226 (11)	1983 (100)
Multiple birth, *n* (%)	443 (22)	1983 (100)
Apgar score at 5 min <7, *n* (%)	73 (3.7)	1326 (67)
*Parental factors*		
Maternal educational level <12 years, *n* (%)	1407 (71)	1973 (99.5)
Pre-existing maternal obesity (BMI > 30 kg/m^2^), *n* (%)	94 (4.7)	770 (39)
Maternal smoking during pregnancy, *n* (%)	297 (15)	1338 (68)
*Growth (0–2 years)*		
Weight for height z score 1 year, mean (SD)	−0.08 (1.09)	1260 (64)
Weight for height z score 2 years, mean (SD)	−0.24 (1.06)	1206 (61)
*Developmental milestones (0–2 years)*		
Smiling, onset age in weeks, mean (SD)	8.5 (3.51)	540 (27)
Speaking 2 to 3 word sentences, *n* abnormal (%)	287 (15)	1382 (70)
Head lifting, *n* abnormal (%)	335 (17)	1019 (51)
Standing, *n* abnormal (%)	353 (18)	1088 (55)
Walking, onset age in months, mean (SD)	15.5 (3.12)	1239 (63)

LOLLIPOP: Longitudinal Preterm Outcome Project, SD: Standard Deviation, *N*: number, SGA: small-for-gestational age, BMI: Body Mass Index.

**Table 2:** Prediction model to identify children at risk of future developmental delay at age 4 according to the ASQ.

Predictor	Categories or Unit of Measurement	Regression Coefficient	OR	95%-CI of OR
Sex	0 = female, 1 = male	1.2	3.5	2.3–5.3
Maternal educational level	0 = 12+ years, 1 = <12 years	0.8	2.3	1.4–3.8
Maternal pre-existing obesity	0 = BMI <30 kg/m^2^, 1 = BMI >30 kg/m^2^	0.6	1.9	0.9–4.0
Smiling	Age in weeks	0.1	1.1	1.0–1.2
Speaking 2–3 word sentences (2 years)	0 = yes, 1 = no	1.7	5.5	3.4–8.7
Standing (1 year)	0 = yes, 1 = no	0.9	2.5	1.4–4.4
Weight for height 1 year	z score	−0.2	0.8	0.7–1.0

OR: odds ratio, 95%-CI: 95% confidence interval. According to the Akaike Information Criterion, a *p*-value < 0.157 is considered statistically significant, and these variables are considered an added value to the prediction model. Each coefficient is multiplied with the shrinkage factor of 0.9748, and subsequently, the new intercept of −5.99 was determined for the shrunken model (pooled optimism factor 0.1181). The linear predictor of this model is −5.99 + 1.21 × Sex + 0.80 × Maternal educational level + 0.60 × Maternal pre-existing obesity + 0.10 × Smiling + 1.65 × Speaking 2–3 word sentences + 0.88 × Standing − 0.21 × BMI z score 1 year.

**Table 3:** Sensitivity, specificity, NPV and PPV for the developed prediction model, operationalized in the calculator, for an abnormal ASQ total score at age 4 at several cut-off values.

Cut-off value	Sensitivity (%)	Specificity (%)	NPV (%)	PPV (%)
Probability 5%	86	62	18	98
Probability 10%	73	80	27	97
Probability 20%	55	91	38	95
Probability 30%	38	96	47	94
Probability 40%	25	98	51	93
Probability 50%	14	99	55	92

Probability: the probability of an abnormal ASQ total score at age four using the linear predictor of the prediction model. NPV: negative predictive value; PPV: positive predictive value.

**Table 4:** Example of the use of the calculator in Excel format, using the developed prediction model for a hypothetical child as described in the results (“*Example*”).

Variable	Unit	
Weight for height z score 1 year	z score	−0.09
Smiling	Age in weeks	13
Maternal pre-existing obesity	0 = no (BMI < 30), 1 = yes (BMI > 30)	1
Maternal educational level	0 = 12+ years, 1 = <12 years	1
Standing	0 = yes, 1 = no	1
Sex	0 = female, 1 = male	1
Speaking 2–3 word sentences	0 = yes, 1 = no	1
	Probability (%)	62.68
